# Attitudinal and Behavioral Inference in Social Influence: A Process Model of Direct and Indirect Judgments and Decisions

**DOI:** 10.3390/bs16071188

**Published:** 2026-07-14

**Authors:** Yubo Zhou, Dolores Albarracín

**Affiliations:** 1 Department of Psychology, University of Pennsylvania, Philadelphia, PA 19104, USA; 2 Annenberg Public Policy Center, University of Pennsylvania, Philadelphia, PA 19104, USA; 3 Annenberg School for Communication, University of Pennsylvania, Philadelphia, PA 19104, USA

**Keywords:** inference, behavioral information, attitudinal information, attitude, norm, persuasion, social influence, behavior relevance, behavior control, processing ability

## Abstract

Psychologists and other behavioral and social scientists have long examined how others’ attitudes and behaviors influence a person’s decisions, attempting to establish the relative impact of attitudinal and behavioral information in persuasion and social influence. However, these comparisons have not always considered how recipients process attitudinal or behavioral information, leading to inconsistent findings. Both attitudinal and behavioral information can inform direct judgments and prompt recipients to draw inferences that influence judgments and decisions. These processes are governed by the processing goals and ability of information recipients and the characteristics of the information.

## 1. Introduction

Although people’s attitudes and behaviors often leave traces in the world (Cialdini et al., 1990), what others say is also a critical source of information. Consider a scenario in which an individual wants to convince others to use an environmentally friendly product. To that end, this individual might state that they *like* the product. They might also state that they *use* the product. The first statement comprises attitudinal information, which involves evaluation of an object (Albarracín & Johnson, 2018; Fazio et al., 1986), whereas the second comprises behavioral information, which describes an overt action or inaction (Albarracín et al., 2018; Dai et al., 2023; Zhou & Albarracín, 2026). A similar distinction appears in the social norms literature pertaining to the attitude or behavior of a group. Information about others’ approval or disapproval, which is conceptually equivalent to attitudinal information, is referred to as injunctive norms, whereas information about the frequency of others’ behavior, which is conceptually equivalent to behavioral information, is referred to as descriptive norms (for injunctive and descriptive norms, see Cialdini et al., 1990, 1991).

Attitudinal and behavioral information can sway recipients because it is persuasive in and of itself. In addition, inferences will likely come into play as well. Learning that someone uses a product may lead recipients to infer that the person likes it, and learning that someone likes a product may also lead to the inference that the person uses it. Thus, to understand persuasion and social influence, it is necessary to characterize the effect of the literal implications of attitudinal and behavioral information, the inferences recipients make, and the conditions and effects of these psychological processes. This paper’s aim is to advance this understanding by reviewing the existing literature against a new set of theoretical hypotheses.

Statements that include both attitudinal and behavioral information are typically more persuasive than either alone (Zhou & Albarracín, 2026). However, audiences may have either attitudinal or behavioral information. For instance, someone may express their attitudes without mentioning how they act, or may display a behavior without disclosing their attitude. In these cases, audiences may still be influenced by the single piece of information that is presented. But precisely how? To answer this question, this paper presents an original theoretical model of how attitudinal or behavioral information about an individual or group influences the audience’s own judgments (i.e., their own attitudes, defined as evaluations) and decisions (i.e., a choice between two alternatives or a behavioral intention, which is the willingness to perform a behavior) when only attitudinal or behavioral information, but not both, is available. Specifically, we bring to bear fundamental social cognitive principles—information processing goals and processing ability (Wyer & Srull, 1994)—to explain how attitudinal and behavioral information about individuals or groups influences the judgments and decisions of an audience. We conceptualize the influence of the presented information and its indirect inferences, integrating previously conflicting findings. In this way, we review existing experiments but remain speculative because no prior research has tested the model’s predictions.

Despite past attention to these issues, prior research has yielded conflicting findings about the relative influence of others’ attitudes vs. others’ behaviors. For example, Tu and Fishbach (2015) compared the effects of attitudinal and behavioral information (i.e., *preference* vs. *action* conditions) on individuals’ behavioral decisions. Participants who learned about others’ preferences, such as someone liking a particular flavor of chewing gum, were more likely to make the same choices than were those who learned about others’ actions or behavioral intentions (Experiment 1; Tu & Fishbach, 2015).^1^ However, other research has found the opposite pattern. In fact, in a study of environmental decisions, participants who learned that others had installed a solar panel were more likely to want to do the same than those who learned that others merely supported solar energy (Kraft-Todd et al., 2018).

One difference between Tu and Fishbach’s (2015) and Kraft-Todd et al.’s (2018) results may lie in the processing goals of the information recipients. For example, Tu and Fishbach studied laboratory interactions in which peers discussed candy preferences or were exposed to surveys about food products (e.g., Swiss Miss Hot Chocolate). In turn, peer interactions and hedonic products may motivate individuals to understand their peers’ attitudes. When two people are getting acquainted, finding out about their likes and dislikes—attitudinal information—is key to forming a connection. Also, products such as hot chocolate are typically evaluated for their hedonic value, making attitudinal information relevant to processing goals and thus influential (for information about object functions, see Shavitt, 1990). In contrast, Kraft-Todd et al.’s solar panels may raise practical considerations about cost and installation. In that case, people who receive information about others’ attitudes or behaviors may be motivated to focus on the others’ behavior more than their attitudes. However, neither the processing goals prompted by the interaction nor the object’s functions have been examined in relation to the influence of attitudinal and behavioral information.

What further complicates the picture is that the goals of information recipients may also affect their *inferences* about others’ attitudes or behaviors. Inference is a cognitive process by which people derive a conclusion from available premises, using internal rules that govern how information is processed (Hastie, 1983), often requiring processing ability (Gottfredson, 1997). For example, people who wonder about someone’s behavior may infer behavior from information about the person’s attitude. We define *behavioral inferences* as assumptions about others’ behavior based on their attitudes and *attitudinal inferences* as assumptions about others’ attitudes based on their behavior. These inferences are forms of attributional reasoning, where people seek to explain the cause of a state or event (for attribution theory, see Gilbert & Malone, 1995; Heider, 1958; Jones & Davis, 1965; Jones & Harris, 1967; Kelley, 1973, 1987; Ross, 1977; Weiner, 1985; Zhou & Albarracín, 2026). However, attribution research has focused on judgments about an actor’s personality or ability, as well as the contextual causes of an actor’s behavior (e.g., luck), without analyzing attributions for other people’s attitudes or behaviors. Moreover, attribution research has focused solely on judgments about an actor rather than on the influence of attributions about others on the observer’s own decisions. Thus, these differences also make our contributions novel.

**Figure 1:**
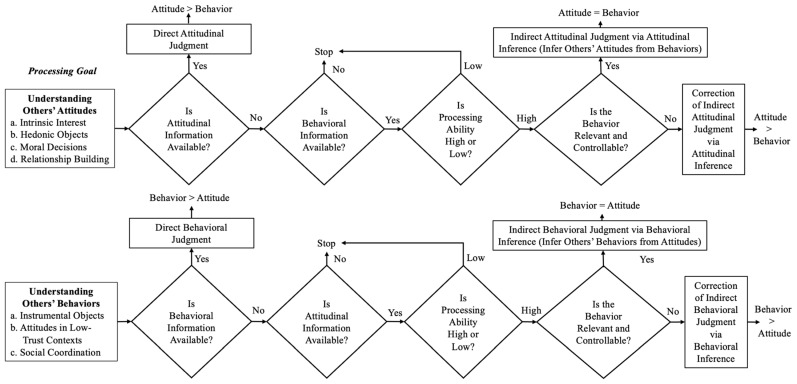
Social influence processing model to understand the impact of others’ attitudes and behaviors.

The model we propose in this paper is presented in Figure 1, and the relevant definitions are shown in Table 1. According to the model, the relative influence of attitudinal and behavioral information depends on recipients’ processing goals, processing ability, and contextual factors such as behavior relevance and controllability. The person’s processing goals may be satisfied through direct judgments or indirect inferences from attitudinal and behavioral information. That is, even if an audience begins by focusing on attitudinal or behavioral information, people may later draw inferences about absent information.^2^ In addition, processing ability may determine whether attitudinal and behavioral inferences occur and whether these inferences are subsequently maintained or corrected based on the relevance of an attitude to a behavior and the controllability of the behavior. As a result, the model generates predictions about the relative influence of attitudinal and behavioral information on the recipients’ own judgments and decisions under different ability conditions. In the figure, a prediction of *Attitude* = *Behavior* indicates that the two types of information exert comparable influence on the recipients’ own judgments and decisions. A prediction of *Attitude* > *Behavior* indicates a stronger influence of attitudinal versus behavioral information, whereas a prediction of *Behavior* > *Attitude* indicates a stronger influence of behavioral versus attitudinal information. If neither attitudinal nor behavioral information is available, or if recipients lack sufficient processing ability to make goal-relevant inferences, no judgment is made, as indicated by the *Stop* endpoint in the figure.

Although we already discussed the differences between our model and attribution theory, other distinctions are in order. To begin, even though concepts like *behavioral control* have been used in the reasoned action approach (Ajzen & Fishbein, 1980; Ajzen et al., 2018; Albarracín et al., 2001), Ajzen and Fishbein were not concerned with inferences about either the self or other people. The reasoned action approach simply uses perceived control as a predictor of *behavioral execution*. Second, even though norm researchers have called attention to behavioral and attitudinal inferences (Deutchman et al., 2025; Eriksson et al., 2015), they have done so without detailing the different processes and outcomes in Figure 1. Moreover, they have not distinguished the attitudinal and behavioral components of injunctive and descriptive norm measures. For example, injunctive and descriptive norm information sometimes contains only attitudinal or behavioral information. A descriptive norm may be manipulated as a bathroom poster stating that most users turn off the lights when leaving a restroom (Leoniak & Cwalina, 2019), whereas an injunctive norm manipulation may indicate that “73% of respondents approved the attitude expressed by female college students that one’s natural and healthy appearance is the most beautiful and needs no change” (Li, 2022). In other cases, however, injunctive or descriptive norm information contains both attitudinal and behavioral information. For example, an injunctive norm message stating that “97% of households think it is important for the environment that households participate in the municipality’s recycling program” conveys both approval of the behavior and the behavior itself (Fuhrmann-Riebel et al., 2024). Thus, even though injunctive and descriptive norms share similarities with attitudinal and behavioral information, our definitions bring greater precision and may thus inform future manipulations and measures.

Processing ability drives cognitive processing in many theories of social cognition and persuasion (Albarracín, 2002; Chaiken, 1980). Some of these models have proposed that qualitatively different processes exist for higher and lower levels of processing ability (e.g., Petty & Cacioppo, 1986). Our model, however, simply characterizes how similar judgments can be made depending on direct or inferred evidence, without distinguishing those processes or the resulting judgments. Moreover, in our model, greater processing ability is conducive to “filling in” judgments based on inferred information, leading to objectively incorrect judgments. Therefore, rather than characterizing our proposal as a *dual-process model*, we prefer to describe it as a *social-cognitive model*.

### Overview of the Model

With respect to people’s processing goals, drawing inferences from others’ attitudes or behaviors and using these inferences as a basis for one’s own decisions is consistent with individuals’ curiosity about others’ minds and behaviors (Epley, 2008; Ickes, 2003), as well as their need to learn from and coordinate with others (Bandura, 1977; Cialdini et al., 1991, 1990). Attitudes and behaviors are linked in the real world because people’s attitudes often predict their behaviors (e.g., Fazio et al., 1982; Fishbein & Ajzen, 1975; Glasman & Albarracín, 2006) and individuals infer their own attitudes from their behaviors (Albarracín & Wyer, 2001; Bem, 1972; Zanna et al., 1981). This suggests that behavioral and attitudinal inferences may be important beyond the self, in the context of perceptions of others. Accordingly, people infer the moral implications of a behavior from its frequency within a group, while also inferring frequency within a group from its morality (Deutchman et al., 2025; Eriksson et al., 2015). However, the social-cognitive dynamics of these inferences have not been conceptualized to date, nor have their effects on the recipients of attitudinal and behavioral information. How these inferences apply to attitudinal and behavioral information about individuals also remains an open question.

In addition to the goals of information recipients, whether direct judgments or indirect inferences drive judgments and decisions should depend on recipients’ processing ability and properties (see Figure 1). Recipients of behavioral information may have the goal of understanding others’ attitudes because they have an intrinsic interest in others’ mental states, need to make hedonic or moral decisions, or want to improve their social relationships. Recipients of attitudinal information may have the goal of understanding others’ behavior because they have to make an instrumental decision, want to assess attitudes others conceal, or need to coordinate their behavior with others (see Figure 1).

Processing ability refers to the availability of cognitive resources, such as attention, required to make an inference (Chaiken, 1980; Wyer & Srull, 1994). Temporary fatigue and cognitive load generally reduce the capacity to elaborate on information (e.g., Kunasegaran et al., 2023; Pessiglione et al., 2025) and may reduce inferences about others’ attitudes or behaviors. These factors may also limit the evaluation of the premises of these inferences to determine whether to incorporate them into a judgment or decision or correct for their influence. However, limited processing ability is unlikely to completely eliminate inferences that are automatic (e.g., Winter & Uleman, 1984; Uleman et al., 1996). For example, when people learn that a behavior is socially approved, they may automatically infer that it is commonly performed (Eriksson et al., 2015). Generating such an inference does not necessarily require substantial cognitive resources, though deliberatively evaluating its validity does. Stimuli in the external environment, such as noise or conversations, can also decrease processing ability and cause people to lose track of the inference in working memory (e.g., Colle & Welsh, 1976; Salamé & Baddeley, 1982). Even after the distraction disappears, people may be unable to recover the thoughts they were processing before the interruption (e.g., Gillie & Broadbent, 1989). Therefore, when the goal is to understand others’ attitudes, but only behavioral information is available, only individuals with sufficient cognitive resources may base their judgments on attitudinal inferences derived from behavioral information after deliberatively evaluating the validity of those inferences.

As shown in Figure 1, given the goal to understand others’ attitudes and high processing ability, one may not only use direct attitudinal (vs. behavioral) information but also draw indirect behavioral or attitudinal inferences. Given sufficient ability, people may draw behavioral inferences from attitudinal information. However, processing ability may also highlight the relevance and controllability of the behavior. Relevance implies that someone will make inferences only when an attitude and a behavior are mentally represented in relation to one another (Glasman & Albarracín, 2006). Control refers to the extent to which a behavior is easy to perform and up to the individual to execute (Ajzen & Madden, 1986). When a behavior is perceived as highly controllable, we propose that people are more likely to expect that a favorable attitude will translate into action.

Regarding relevance, others’ attitudes may not always seem relevant to the judgment a recipient is trying to make. Learning that someone likes bagels may not seem relevant to determining if that person bakes bagels. By the same token, when someone states that they like a product, others may infer that the person has used it, not manufactured it. The greater the relevance of attitudes to behaviors, the more readily people should draw behavioral inferences from attitudinal information and attitudinal inferences from behavioral information (for the concept of behavioral relevance in the attitude-behavior relation, see Glasman & Albarracín, 2006). However, the perceived correspondence between attitudes and behaviors can vary across cultures. In collectivistic cultures, where people are more likely to consider others’ attitudes, their behavior is less closely aligned with their own attitudes (Markus & Kitayama, 2003; Savani et al., 2008). Accordingly, attitudinal inferences from behavior may be less common, thereby reducing the effect of behavioral information (Barnes & Shavitt, 2023). In contrast, in individualistic cultures, where decisions are often based on personal attitudes, behavioral information might elicit more attitudinal inferences and be more influential (Barnes & Shavitt, 2023).

Controllability can shape individuals’ behavior and should therefore influence an observer’s inferences from behavioral information. Importantly, controllability includes constraints related to financial and temporal resources. People consider their own financial constraints when making behavioral decisions (e.g., Mani et al., 2013), and behavior reflects attitudes only when the actor has sufficient financial resources to carry it out (Ajzen & Madden, 1986; Albarracín et al., 2004). Thus, the cost of an object should influence the extent to which recipients perceive an individual or a group as having control over the behavior. An expensive product may suggest that people have limited control over purchasing it because they depend on scarce financial resources. For example, when someone believes that those who like a luxury car generally cannot afford one, attitudinal information will not yield a behavioral inference, leaving behavioral information to retain its greater influence. By contrast, an inexpensive product may suggest that people have control over purchasing it because they have the necessary financial resources. Under these circumstances, attitudinal information is more likely to produce strong behavioral inferences.

Also, the degree to which an attitude or behavior is perceived as controlled by the individual is likely to affect inferences (for correspondent inference theory; see Jones & Davis, 1965). For example, when a behavior reflects a collective decision, an individual’s action is often perceived as less controllable because it is influenced by the decisions of the group members. As a result, individuals may engage in collective behaviors that do not follow their attitudes. In contrast, individual decisions are generally perceived as more controllable because they are controlled by the individual. Under these circumstances, people’s attitudes are more likely to influence their own behavior, and, therefore, their behavior should provide a strong basis for inferring their underlying attitudes.

In summary, people who have information that directly meets their processing goals will likely use available attitudinal or behavioral information. However, when that information is unavailable, they may make a behavioral or attitudinal inference to reach an indirect judgment (see Figure 1). In making these inferences, individuals should use their cognitive resources to determine whether the inference is valid, and should not proceed when a link between the attitude and the behavior is unjustified. This may occur when the behavior is irrelevant to a particular attitude or when it is difficult to control. As a result, information recipients may judge this indirect behavioral inference as invalid and discount its influence on subsequent judgments. Whether this inference correction reverses the effect of the behavioral information should depend on the strength of the correction (for the flexibility of corrections, see Wegener & Petty, 1997).

This paper presents our model and reviews existing findings as a proof of concept. To explore the model’s explanatory potential, we reviewed a mixed body of persuasion and social influence findings from publicly available empirical experiments. Table 2 shows these experiments according to processing goals, processing ability, and behavior relevance and controllability (see Figure 1).^3^ We identified the processing goals of participants in each experiment based on whether the presented information satisfied recipients’ interest in others’ attitudes (i.e., intrinsic interest), characteristics of the information target (i.e., hedonic objects, instrumental objects, and moral decisions), and social conditions, such as whether a context is low-trust (i.e., attitudes in low-trust contexts) or elicits social coordination. We also identified whether recipients had high (vs. low) processing ability to process the information based on the procedures described in each experiment, designating ability as low when manipulated information was presented alongside unrelated information that could reduce processing capacity.

As shown in Table 2, we also determined the relevance of an attitude to a behavior based on whether the targets of the attitudinal and behavioral information were the same. For example, the behavior most relevant to liking a food is eating that food, rather than selling it or eating a different food. Also, we coded behavioral controllability based on the effort required to perform the behavior, the financial value of the target object, and the involvement of others. Greater effort, higher financial cost, and stronger involvement of others were interpreted as indicating lower controllability. All classifications are based on our interpretation of the procedures and contexts described in the published studies illustrated in Table 2.

In Table 2, we also determined whether an experiment’s findings were consistent with the predictions of our model (see Figure 1) based on the influence of (a) processing goals, (b) processing ability, and (c) behavior relevance and controllability. When no significant difference between attitudinal and behavioral information was observed, we deemed the two as exerting a similar influence.^4^ If an experiment included significant effects and nonsignificant differences across different measures, we flagged the study as supporting both patterns (e.g., > or =). Because the variables proposed by our model have not been explicitly investigated, we were unable to identify examples for every combination of goals, processing ability, relevance, and controllability. As a result, the main contribution of this review was to guide theory building. We also acknowledge that attitudinal and behavioral information is typically not equated in strength. Therefore, the true test of this model awaits future experimentation with better information calibration.

## 2. Perceiver’s Processing Goals

As shown in our model (Figure 1), understanding others’ attitudes vs. behaviors should inform the effects of attitudinal and behavioral information. Accordingly, the goal of understanding someone’s attitudes should increase attention to and inferences about attitudinal information. The goal of understanding someone’s behavior should increase attention to and inferences about behavioral information. In Table 2, the literature is organized according to either of these goals as the first decision point in Figure 1. However, predictions are also based on how processing ability, behavior relevance, and behavior controllability (see Figure 1) might have affected the results from these experiments.

### 2.1. The Goal to Understand Others’ Attitudes

The influence of attitudinal information is likely to depend on the goal of understanding others’ attitudes (see Figure 1). In our review (see Table 2), the goal of understanding attitudes was gauged by (a) intrinsic interest in others’ mental contents, (b) hedonic objects, (c) moral decisions, and (d) the need to strengthen social relationships (see Figure 1).

#### 2.1.1. Intrinsic Interest in Others’ Mental Contents

A primary motivation for understanding others’ attitudes is an intrinsic interest in their mental contents (Premack & Woodruff, 1978). For example, people spend considerable time gossiping and speculating about what others think (e.g., Hartung & Renner, 2011, 2013; Renner, 2006). Not knowing what others think creates an information gap that is experienced as aversive and motivates information seeking (Case et al., 2005; Kellermann & Reynolds, 1990). Attitudinal information about others, or inferences about their attitudes, can thus satisfy one’s curiosity either directly or indirectly (see Figure 1).

As shown in Table 2, the findings of experiments in which participants likely had a stronger intrinsic interest in others’ attitudes were consistent with the predictions of our model. Specifically, Li (2022, Experiment 1) found that information about others’ attitudes toward body satisfaction exerted a stronger influence on female college students’ body satisfaction than information about others’ actual body satisfaction–related behaviors.^5^ As young adult women are intrinsically interested in other women’s body image attitudes (e.g., Fardouly et al., 2015; Tiggemann et al., 2018), the greater influence of attitudinal (vs. behavioral) information is consistent with our model. This pattern also makes sense given that participants in this study might have had low ability, as the experimental procedure included filler questions and a time estimation task that likely increased cognitive load. Thus, even though the attitudes and behaviors investigated (i.e., approval of behaviors related to body satisfaction and dieting) were highly relevant to one another and the behavior was controllable, limited processing ability might have constrained attitudinal inferences from behavioral information.

#### 2.1.2. Hedonic Objects

Understanding others’ attitudes may also be important when an object is hedonic (see Figure 1). By definition, hedonic objects lead someone to focus on the affective experiences they provide, whereas instrumental ones lead people to focus on practical behavioral goals (Batra & Ahtola, 1991; Dhar & Wertenbroch, 2000; Millar & Tesser, 1986). Accordingly, hedonic objects may increase attention to others’ attitudes, whereas instrumental ones may increase attention to others’ behaviors.

Empirical findings consistent with our predictions are illustrated in the hedonic object examples in Table 2. For example, Tu and Fishbach’s (2015; Experiment 1) research showed an advantage of attitudinal information for other hedonic objects such as candy. However, in this experiment, a third party (i.e., the experimenter) related what others had said, possibly introducing a distraction. As a result, attitudinal information about hedonic objects exerted a stronger influence than behavioral information.

Moving to other experiments, Mollen et al. (2021, Experiment 2) found that learning about others’ approval of snacks had more influence on participants’ snack choices than learning about others’ consumption behavior. Given the likely hedonic functions of the snacks, sufficient ability and high relevance and controllability should predict direct judgments from attitudinal information and attitudinal inferences from behavioral information, leading to no difference between the two types of information (see Figure 1). However, processing ability might have been limited by the inclusion of a filler task, namely, evaluating abstract paintings unrelated to the experiment. Thus, our model would predict that attitudinal information would exert a stronger influence than behavioral information. As shown in the table, however, only some of the measures showed this pattern, making the findings only partially consistent with our model’s predictions (see Table 2).

Two additional experiments involved information about hedonic objects (i.e., Starbucks gift cards and local travel guides). However, recipients likely had sufficient processing ability to generate inferences, and the inferable behaviors were both relevant to and controllable by the corresponding attitudes. Under these conditions, the model predicts comparable effects of attitudinal and behavioral information. The findings of one experiment were partially consistent with this prediction (Barnes & Shavitt, 2023; Experiment 1), whereas the findings of the other were fully consistent with our model (Hardeman et al., 2017; Experiment 3).^6^

#### 2.1.3. Moral Decisions

Understanding others’ attitudes may also be the goal when a behavior is morally relevant (see Figure 1 and Table 2). For example, learning that certain immoral behaviors, such as cheating on a test, are frequent, leads to judgments that the behaviors are approved by a group (Deutchman et al., 2025). Participants who learned that most others engaged in cheating inferred greater social approval of cheating and reported stronger intentions to do the same (Deutchman et al., 2025; Experiment 1). However, this pattern was weaker for fashion decisions such as wearing socks with sandals. In such cases, although participants still inferred people’s attitudes from their behavior, the magnitude of the inferences was substantially smaller. Thus, although this experiment did not compare attitudinal and behavioral information, the findings are suggestive.

Table 2 also presents three experiments that compared attitudinal and behavioral information about moral issues and are consistent with our model’s predictions. Although attitudinal information may exert a stronger influence than behavioral information when people make direct attitudinal judgments, high processing ability, along with relevance and controllability, may enable attitudinal inferences from behavioral information. When that happens, the effects of attitudinal and behavioral information may be similar. Accordingly, in a study of ethical behavior (Liu, 2023; Experiment 1), children were less likely to lie about a forbidden behavior when they learned that other children approved of honesty (attitudinal information) than when they learned about other children’s honest behavior. Given that children likely had sufficient processing ability when receiving the information, and that the attitudinal information was relevant to the behavior and the behavior was controllable, these children likely generated behavioral inferences from attitudinal information. Given these behavioral inferences, attitudinal and behavioral information exerted comparable influence. The other two experiments had similar ability, relevance, and controllability conditions and showed the same pattern (i.e., Dimant et al., 2020; Experiment 1; Ma & Nan, 2018; Experiment 1).

#### 2.1.4. The Goal to Build Relationships

Understanding others’ attitudes should also serve the goal of building relationships (see Figure 1). First, individuals are likely to consider others’ attitudes when deciding whether to initiate a relationship. People prefer others who share similar attitudes and avoid those who hold opposing views (e.g., Singh et al., 2017; Zorn et al., 2022). Second, those seeking close relationships, such as friendships or romantic partnerships, carefully attend to others’ attitudes. Furthermore, a willingness to share attitudes with others can signal mutual trust and affirm the relationship. Therefore, those seeking to deepen a relationship may attend to the others’ willingness to share attitudes, particularly on sensitive topics, as an indicator of relational closeness. For example, individuals in romantic relationships inquire more frequently about each other’s attitudes than those who are merely acquaintances, thereby achieving an intimate understanding of one another (e.g., Berg & Clark, 1986). Although no research on the influence of attitudinal and behavioral information has examined strangers vs. intimates as information sources, our model suggests that attitudinal information should exert a stronger influence in close relationships.

As shown in Table 2, three of the four experiments in which relationship building was important reported results consistent with the predictions of our model. In cases where recipients likely had limited processing ability to fully generate inferences, or where generated inferences were unjustified because the behavior was low in controllability, attitudinal information exerted a stronger influence than behavioral information. For example, in an experiment on college drinking (Goode et al., 2014; Experiment 2), students drank less when they learned that their sorority members disapproved of excessive drinking than when they learned that their sorority members did not drink. College students in a sorority may have greater motivation for social coordination and a greater need to maintain social bonds. At the same time, quitting drinking often requires substantial personal effort, making the behavior relatively low in controllability. Therefore, when individuals have sufficient processing ability to make attitudinal inferences from others’ behavior, the indirect attitudinal judgments resulting from these inferences may be corrected and consequently exert less influence.

Another experiment where relationship building was deemed important showed an advantage of attitudinal information. The experiment, which involved low ability and high behavior controllability, emphasized the communicator’s identity as a friend (e.g., “Suppose a friend of yours is standing next to you.”; Tu & Fishbach, 2015; Experiment 5), thus activating a relationship goal. In the same experiment, the advantage of attitudinal over behavioral information disappeared when the communicator was described as a stranger.

The results of two other experiments showed comparable effects of attitudinal and behavioral information. One experiment compared neighbors expressing positive attitudes toward energy-efficient houses with neighbors’ related behaviors (Belaïd & Flambard, 2023; Experiment 1). Although relationship-building goals should prioritize attitudinal information, recipients likely had sufficient processing ability to draw attitudinal inferences from behavioral information. Accordingly, in the absence of low behavior relevance or low control, the model predicts comparable effects of attitudinal and behavioral information, which was supported by the findings.

Another experiment focused on quitting alcohol, a behavior that typically requires substantial effort and therefore may be relatively low in controllability (Wheeler, 2018; Experiment 1). Because the behavior was relatively difficult to control, the model predicts that behavioral information should be discounted and attitudinal information should retain its stronger influence. Contrary to this prediction, however, the results showed no significant difference between the effects of attitudinal and behavioral information.

### 2.2. The Goal to Understand Others’ Behavior

As shown in Figure 1, people may seek to understand others’ behavior for various reasons, including factors related to the decision or the context. In Table 2, the goal to understand behavior was identified based on involving (a) instrumental objects, (b) an interest in others’ attitudes in low-trust contexts, and (c) social coordination (see Figure 1).

#### 2.2.1. Instrumental Objects

As discussed for the goal to understand others’ attitudes, people are interested in others’ mental states, such as their evaluations of objects. However, whereas attitudes are particularly important for hedonic objects, instrumental objects may call attention to behavioral information, such as frequency of use. For example, people are more interested in whether a solar panel effectively provides clean energy. As a result, whether others have acquired solar panels may become more relevant and therefore more influential in shaping behavioral decisions.

As shown in Table 2, all three experiments involving instrumental objects were consistent or partially consistent with our model’s predictions. In one experiment, participants were more likely to choose a computer when they learned about others’ attitudes than when they learned about others’ computer use (Trujillo et al., 2021, Experiment 1A). Since participants in these experiments likely had high processing ability and the behaviors were both relevant to the attitudes and controllable, our model predicts that strong behavioral inferences could approximate the effect of the attitudinal information. Accordingly, the findings were consistent with this prediction.

Another experiment manipulated attitudinal and behavioral information in a group discussion about an instrumental object (i.e., a computer), finding that behavioral information exerted a stronger influence than attitudinal information (Zhou & Albarracín, 2023; Experiment 3). This finding is consistent with our model’s predictions, as information presented by multiple communicators may constrain processing ability. Under such conditions, recipients may rely more on direct judgments aligned with their goal of understanding others’ behavior, resulting in a stronger influence of behavioral than attitudinal information. The only experiment that produced results that were only partially consistent with our model manipulated attitudinal and behavioral information about probiotic supplements, which are typically consumed to achieve instrumental goals, such as improving health, rather than to satisfy hedonic goals, such as seeking pleasure (Zhao et al., 2023, Experiment 1). Consistent with our predictions for a study with high ability and high behavior relevance and controllability, some experimental measures showed no significant difference between attitudinal and behavioral information. Nonetheless, other measures showed a stronger influence of the behavioral information.

In summary, we identified three experiments involving instrumental objects, including cases in which recipients likely had relatively high or relatively low processing ability. The findings across these experiments were consistent or partially consistent with our model’s predictions. When processing ability is low, people are more likely to base their decisions directly on behavioral rather than attitudinal information. When processing ability is high, they may draw behavioral inferences from attitudinal information and use them in their decisions, as the behavior is relevant to and controllable by the corresponding attitudes. Nonetheless, the experiments in Table 2 were not designed to test the influence of these factors. A proper examination of these effects is only possible with better-calibrated manipulations of attitudinal and behavioral information, as well as of goal strength, processing ability, behavior relevance, and behavior controllability.

#### 2.2.2. Attitudes in Low-Trust Contexts

Others’ behavioral information may also be used to infer their attitudes when trust in them is low (see Figure 1). Demonstrating a behavior typically entails higher costs than expressing an attitude, making it a more credible signal (for signaling theory, see Connelly et al., 2011). Compared with attitudes, behaviors are sometimes perceived as reliable, difficult-to-conceal indicators of internal states (for research on credibility-enhancing display, see, e.g., Henrich, 2009; Kraft-Todd et al., 2018; Lanman & Buhrmester, 2017; Turpin & Willard, 2022). Consequently, when individuals suspect that others may hide their true attitudes, their behavior may serve as trustworthy evidence of them.

As shown in Table 2, two experiments involved contexts in which behavioral information could serve as a more reliable indicator of attitudes than attitudinal information itself because ability was high, behavior relevance was high, and behavior controllability was low. For example, in Kraft-Todd et al. (2018; Experiment 2), installing solar panels requires substantial financial investment, making it unreasonable to infer corresponding behavior solely from others’ attitudes. People may express positive attitudes toward an expensive product but choose not to purchase or use it because of economic constraints. As a result, such behavioral inferences based on attitudes may be corrected and therefore exert little influence on decisions.

Another experiment found no significant difference in alcohol consumption frequency between participants who recalled others’ attitudes toward drinking alcohol and those who recalled others’ actual drinking behavior (Lac & Donaldson, 2020; Experiment 1). As mentioned, reducing alcohol drinking requires substantial effort, making its controllability low. Under these conditions, our model predicts that behavioral information should exert a stronger influence than attitudinal information. However, the findings were inconsistent with this prediction.

#### 2.2.3. The Goal to Coordinate with Others

An important motivation for attending to others’ behavior is the need to coordinate with others, which includes cooperating to avoid social sanctions and competing to secure desired outcomes. People can infer others’ internal mental states from their behavior, thereby facilitating more effective social interactions (Kanske & Murray, 2019). They also mimic others’ behaviors (Chartrand & Bargh, 1999) and coordinate their contributions in a public goods situation (Cialdini et al., 2006; Fischbacher et al., 2001). As a result, our model predicts that the need to coordinate behavior will generally lead people to focus on others’ behavior (see Figure 1).

As shown in Table 2, one experiment that illustrated social coordination goals was partially consistent with our model’s prediction. Informing participants that others intervene to help victims of abusive relationships (behavioral information) had more influence on intentions to help than informing them that others approved of helping (attitudinal information; Lemay et al. 2019). Although participants likely had sufficient processing ability to draw behavioral inferences from attitudinal information, helping a victim is often low in controllability, which should make participants more cautious in making these inferences. Accordingly, behavioral information had more influence than attitudinal information.

## 3. Conclusions

As shown in Figure 1, this paper proposes a theory that distinguishes between direct judgments based on available attitudinal or behavioral information and indirect judgments formed through inferences about others’ attitudes and behaviors in social influence. People can reach a decision by observing that others approve of an issue or engage in a behavior. They can also make decisions after inferring others’ behaviors from their attitudes or others’ attitudes from their behaviors. Moreover, people focus on attitudinal or behavioral information depending on their goals, such as curiosity about others’ attitudes or behavior, the need to form and maintain relationships, and/or the desire to coordinate one’s behavior with others (see Figure 1). Individuals may then encode the available attitudinal or behavioral information and make a direct judgment without engaging in a more complex inference process.

In addition to proposing a theoretical model, as summarized in Table 2, we reviewed prior experiments on the relative influence of attitudinal and behavioral information and classified the studies within each report according to the factors proposed in our model. This review allows us to examine whether patterns in previous findings provide preliminary support for our model’s predictions. The experiments in Table 2 are broadly consistent with our model. However, the experiments we reviewed are not exhaustive, and we did not estimate the magnitude of the different effects implied by the model. Thus, our work should be furthered through future experimental tests of these predictions. Future research should test our model more directly and conduct a meta-analysis of all available data.

Inferences are likely when people have high cognitive resources. The process of making an inference also depends on properties of the behavioral and attitudinal information. In particular, the inference process is governed by two principles: relevance and control. Relevance concerns whether attitudinal information is sufficiently related to a corresponding behavior to activate an attitude–behavior association in support of the inference. Control concerns whether behaviors are under a person’s will, as controllable behaviors can follow and signal a person’s attitudes more than uncontrollable ones. Neither of these factors has been investigated experimentally, leaving an area that is ripe for future research.

As shown in Figure 1, behavior control is key to behavioral and attitudinal inferences. Because moral behaviors are often socially desirable, individuals may perform them under external pressure rather than out of personal endorsement. As a result, attitudinal information may be particularly important in these cases. Certain hedonic and luxury objects may operate similarly. People may purchase luxury goods for social reasons, weakening the expected correspondence between attitudes and behaviors. In those cases, attitudinal information may be particularly informative.

Perceived controllability is an important consideration in our model and can include a behavior’s cost. When a behavior involves substantial costs, such as purchasing an expensive product, individuals may hold favorable attitudes toward the object but still be unable or unwilling to act due to limited financial resources. In such cases, observers may now draw or later correct attitude-based behavioral inferences because the behavior is not fully under the actor’s control. At the same time, engaging in costly behaviors often signals a stronger commitment to the object, making such behaviors a more reliable basis for drawing attitudinal inferences. Thus, perceived controllability may also influence the strength and validity of attitudinal and behavioral inferences, thereby shaping the relative influence of attitudinal and behavioral information in complex ways.

The model we proposed offers a new perspective on puzzling findings that have accumulated over the past decade (see Table 2). The inconsistency in past findings probably stems from a lack of attention to participants’ goals at the time they received the information, processing ability, whether the available information suggests a link between attitudes and behaviors, or whether the behavior is under the person’s control. Moreover, past studies have measured direct judgments without accounting for the role of inferences in shaping behavioral decisions. People might approach information with different processing goals, such as an interest in others’ attitudes or a desire to understand others’ true views about an object. These differing goals can lead individuals to either rely on direct judgments or on inferences. Future research should directly manipulate the inferences people draw and compare the conditions that elicit different inferences.

### 3.1. Limitations

We discussed how goals shape both the direct judgments people make from attitudinal and behavioral information and the indirect judgments arising from inferences about missing information (e.g., drawing attitudinal inferences from behavioral information). We also presented hypotheses about how these processes, along with processing ability, influence decisions. We further analyzed cases in which behaviors are difficult to control, discussing how this perception may alter inferences and, in turn, change the relative influence of attitudinal and behavioral information on recipients’ own judgments and decisions. However, as shown in Figure 1, relevance, like controllability, should also determine inferences, yet Table 2 includes only cases involving relevant attitudes and behaviors. This situation is not surprising because existing experiments comparing the influence of attitudinal and behavioral information have not directly manipulated relevance. As a result, although our paper conceptualizes why relevance and controllability should determine the influence of attitudinal and behavioral inferences on recipients’ judgments and decisions, the existing literature has not empirically evaluated how irrelevant or uncontrollable behaviors, relative to relevant and controllable behaviors, alter these inferences and their downstream effects on recipients’ own judgments and decisions. This limitation largely reflects the fact that prior research has focused on the relative influence of attitudinal and behavioral information rather than inferential processes, let alone the dynamics of these inferences. A careful analysis of these processes will require directly manipulating relevance and controllability in the future.

Although our review was not systematic, the results in Table 2 are highly suggestive, indicating full and partial support of all predictions. However, key predictions about the equal influence of attitudinal and behavioral information must be empirically tested relative to a baseline. However, the experiments conducted to date lacked proper baselines. Moreover, predictions from our model must be refined by specifying when a particular factor might carry the day. For example, certain interpersonal goals might render attitudinal information irreplaceable, while other factors, such as behavioral controllability, might have stronger or weaker effects in different contexts. Thus, future research should consider these possibilities.

Finally, two experiments involving alcohol drinking and high ability showed no difference between attitudinal and behavioral information, even though the model predicted a difference. However, the predictions in Table 2 rely on specific conditions that we cannot verify, as the past experiments neither controlled nor manipulated them. Therefore, only experimental manipulations of factors such as ability or controllability of behavior will test our predictions.

### 3.2. Future Directions

All of this said, several directions not addressed in the current paper warrant attention. To begin, we did not address whether attitudinal or behavioral information is more persuasive when the two conflict. Although such conflict can reduce the overall effectiveness of the information (e.g., Kraft-Todd et al., 2018), the audience’s goals should determine the information’s impact. When the goal is to understand others’ attitudes, the attitudinal information itself, or attitudinal inferences derived from behavioral information, should have a stronger influence on decisions than the behavioral information. Conversely, when the goal is to understand others’ behavior, behavioral information itself, or behavioral inferences derived from attitudinal information, should exert a greater influence. Whether this prediction holds up is an open question.

Another important issue concerns how different types of attitudinal and behavioral information influence inferences and decisions. Specifically, behavioral or attitudinal inferences derived from general versus specific attitudes and behaviors may differ in the scope of behaviors and attitudes they affect. General attitudes and behaviors may support broader inferences across a range of behaviors or attitudes, whereas specific attitudes or behaviors may exert a stronger influence on those that are most closely aligned with them. Using attitudinal information as an example, when individuals express a general attitude, such as liking modern technology, others may infer that they are more likely to try new artificial intelligence models and electric vehicles. In contrast, specific attitudes are less likely to generate inferences about unrelated behaviors, such as whether a person who likes satellites will use electric vehicles.

Repetition of information is another factor not covered by our model that may influence the relative impact of attitudinal and behavioral information on recipients’ behavior. Repetition of persuasive information has been shown to produce a curvilinear effect on its persuasive impact. As exposure frequency increases, the influence of the information initially strengthens, then declines (Cacioppo & Petty, 1979). For example, repeated expressions of a positive attitude toward an object may signal confidence in that attitude, thereby increasing its impact (for attitude strength, see Krosnick & Petty, 2014). However, repeated expression of an attitude may be interpreted as an attempt at persuasion, thus increasing psychological reactance.

Cultural differences may also shape the extent to which people infer that others’ behaviors are driven by their attitudes. However, none of the studies we reviewed directly compared the extent to which people from different cultures draw inferences from attitudinal information and behavioral information. Nevertheless, findings from related research allow us to consider how cultural differences may influence these inferences and, in turn, indirectly shape behavioral decisions. As mentioned, in collectivistic cultures, decisions are often influenced by factors beyond personal preferences, such as social obligations and others’ expectations, to a greater extent than in individualistic cultures (Eom et al., 2016; Markus & Kitayama, 2003; Savani et al., 2008). For example, Americans are more likely to choose objects they personally prefer, whereas Indians are more likely to consider family preferences (Savani et al., 2008). This observation has important implications for our model. Specifically, individuals from collectivistic cultures may be less likely to view others’ attitudes and behaviors as tightly connected. As a result, information about others’ attitudes or behaviors may be less likely to elicit behavioral or attitudinal inferences among individuals from collectivistic versus individualistic cultures. Although some preliminary evidence suggests that the influence of others’ attitudes and behaviors differs across cultures (Barnes & Shavitt, 2023), no research has directly tested whether such differences arise through cultural variation in attitudinal or behavioral inferences. Future research should therefore consider the potential role of culture.

In closing, the extent to which recipients use attitudinal or behavioral information affects their behavioral decisions. Existing empirical evidence has begun to document behavioral inferences from attitudes and attitudinal inferences from behavior (Deutchman et al., 2025; Eriksson et al., 2015; Zhou & Albarracín, 2026). However, this work cannot progress without specifying the social-cognitive principles of those inferences and when they occur. Therefore, future empirical tests of our framework are essential for clarifying the processes of social influence and informing its practical applications.

## Figures and Tables

**Table 1:** Definitions of key concepts.

Concept	Definition
Attitude	An overall evaluation of an object, person, or issue made by an individual or group.
Behavior	Any observable action or inaction performed by an individual or group.
Inference	A cognitive process through which conclusions are derived from available information, evidence, or premises using reasoning rules.
Attitudinal Inference	The process of assuming another person’s attitudes based on their observed behavior.
Behavioral Inference	The process of assuming another person’s behavior based on their attitudes.
Judgment	An assessment or conclusion formed about an object, person, event, or situation based on available information and/or personal interpretation. It includes attitudes as well as beliefs, which are probability assessments.
Direct Judgment	Judgments based on others’ stated attitudes as well as stated or directly observable behaviors.
Indirect Judgment	Judgments based on attitudes or behaviors inferred from available information about others.
Decision	A choice made between two or more alternatives, often resulting from evaluating available information and potential outcomes.
Intention	Willingness to perform a behavior.
Processing Goal	A desired outcome that guides the cognitive operations and can be activated by the need to make a judgment or decision.
Intrinsic Interest	People’s inherent desire to know others’ attitudes, even in the absence of any practical benefit.
Hedonic Object	Objects that provide pleasure or other positive emotional experiences to their owners.
Instrumental Object	Objects that possess utilitarian value and provide practical functions or benefits to their owners.
Moral Decision	Choices made on the basis of ethical principles or values regarding what is right or wrong, ethical or unethical, or fair or unfair.
Processing Ability	The mental capacities and resources involved in acquiring, processing, storing, and applying knowledge, including reasoning, memory, and attention.
Relevance	The extent to which an individual’s attitudes are mentally represented as connected to their behavior.
Controllability	The degree to which performing a behavior is perceived to be under an individual’s control (e.g., devoid of external constraints).

**Table 2:** Summary of Articles Reporting Inconsistent Findings on the Relative Impact of Attitudinal and Behavioral Information, Including Processing Goals, Reference, Summary, Processing Ability, Relevance, Control, Model Prediction, Experimental Results, and Model Fit.

Processing Goals	Reference	Summary	Processing Ability	Relevance	Control	Model Prediction	Experiment Results	Model Fit
Goal of Understanding Others’ Attitudes								
**Intrinsic Interest**	Li (2022), Exp 1	Female college students were more likely to report increased body satisfaction when others expressed supportive attitudes toward body satisfaction than when others engaged in supportive body image–related behaviors.	Low	Yes	Yes	Attitude > Behavior	Attitude > Behavior	Yes
**Hedonic Objects**	Mollen et al. (2021), Exp 2	Participants are more likely to consume healthy snacks after learning that others disapprove of unhealthy snacking than learning about others’ actual consumption behavior.	Low	Yes	Yes	Attitude > Behavior	Attitude > or = Behavior	Partial
	Tu and Fishbach (2015), Exp 1	Participants are more likely to choose the chewing gum that others like than the one others say they want to try.	Low	Yes	Yes	Attitude > Behavior	Attitude > Behavior	Yes
	Barnes and Shavitt (2023), Exp 3	Participants with a more interdependent (vs. independent) self-construal are willing to pay more for Starbucks gift cards when Starbucks was described as a brand that most people like, rather than a brand that most people buy.	High	Yes	Yes	Attitude = Behavior	Attitude > or = Behavior	Partial
	Hardeman et al. (2017), Exp 3	Participants who learn that most travelers approve of booking a local guide showed similar level of preference for choosing the guide as participants who learned that most travelers had booked the guide.	High	Yes	Yes	Attitude = Behavior	Attitude = Behavior	Yes
**Moral Decisions**	Liu (2023), Exp 1	Children are less likely to lie when they learn that other children approve of telling the truth, showing similar reductions in lying as when they learn that other children tell the truth.	High	Yes	Yes	Attitude = Behavior	Attitude = Behavior	Yes
	Dimant et al. (2020), Exp 1	Participants are equally likely to behave honestly in tasks that provide opportunities to cheat when they know that others approve of honesty compared to when they know that others behave honestly.	High	Yes	Yes	Attitude = Behavior	Attitude = Behavior	Yes
	Ma and Nan (2018), Exp 1	Participants show more positive attitudes toward people with mental illness when they learn that mental illness is socially accepted than when they learn about others’ behaviors related to mental illness.	High	Yes	Yes	Attitude = Behavior	Attitude = Behavior	Yes
**Relationship Building**	Goode et al. (2014), Exp 2	College students are less likely to consume alcohol when they are informed that sorority members who are like them disapprove of excessive drinking than when they receive information about those members’ excessive drinking behavior.	High	Yes	No	Attitude > Behavior	Attitude > Behavior	Yes
	Tu and Fishbach (2015), Exp 5	Participants are more likely to intend to obtain a consumer product when they learn that a friend likes it than when they learn that the friend uses it.	Low	Yes	Yes	Attitude > Behavior	Attitude > Behavior	Yes
	Belaïd and Flambard (2023), Exp 1	Participants who learned that their neighbors were satisfied with energy-efficient houses did not show a different likelihood of choosing such houses compared with those who learned that their neighbors had chosen them.	High	Yes	Yes	Attitude = Behavior	Attitude = Behavior	Yes
	Wheeler (2018), Exp 1	Participants perceive similar level of peer norms regarding alcohol-related interpersonal violence behaviors when they learn that fraternity members disapprove of the behavior than when they learn about others’ behavior.	High	Yes	No	Attitude > Behavior	Attitude = Behavior	No
Goal of Understanding Others’ Behaviors								
**Instrumental Objects**	Zhou and Albarracín (2023), Exp 3	Participants show stronger intentions to purchase a laptop when they learn about others’ actions related to the product than when they learn about others’ positive attitudes toward it.	Low	Yes	Yes	Behavior > Attitude	Behavior > Attitude	Yes
	Trujillo et al. (2021), Exp 1A	Participants are more likely to choose an eco-friendly notebook when they learn that others approve of using it than when they learn that others use it.	High	Yes	Yes	Behavior = Attitude	Behavior = Attitude	Yes
	Zhao et al. (2023), Exp 1	Participants show stronger intentions to purchase probiotic supplements when they are encouraged to perform an action related to the product, such as asking a healthcare provider about it, than when they are exposed only to evaluations of the supplement.	High	Yes	Yes	Behavior = Attitude	Behavior > or = Attitude	Partial
**Attitudes in Low-Trust Contexts**	Kraft-Todd et al. (2018), Exp 2	Participants are more likely to adopt clean energy when they learn that its promoter uses the product than when they learn about the promoter’s supportive attitudes toward it.	High	Yes	No	Behavior > Attitude	Behavior > Attitude	Yes
	Lac and Donaldson (2020), Exp 1	Participants consume alcohol at similar frequencies when they recall others’ drinking behavior than when they recall others’ approval of drinking alcohol.	High	Yes	No	Behavior > Attitude	Behavior = Attitude	No
**Social Coordination**	Lemay et al. (2019), Exp 1	Participants show stronger intentions to help individuals experiencing abuse when they learn that others act in such situations than when they learn that others hold positive attitudes toward intervening.	High	Yes	No	Behavior > Attitude	Behavior > or = Attitude	Partial

***Note*.** The experiment numbers reported here correspond to the original numbering used in the source articles. The symbol “> or =” in the experiment results indicates that the experiment included multiple decision-related measures, some of which showed significant directional effects, whereas others did not.

## Data Availability

The original contributions presented in this study are included in the article. Further inquiries can be directed to the corresponding authors.

## Notes

1 More specifically, the action condition was someone liking and wanting to try a particular flavor of chewing gum.

2 The absence of ability does not imply that inferences are eliminated entirely. Even when ability and motivation are low, people can still draw trait inferences spontaneously from some’s behaviors (e.g., Uleman et al., 2008, 1996; Winter & Uleman, 1984). For example, observing that a librarian carries someone’s groceries across the street may lead individuals to infer that the librarian is helpful, even in the absence of an explicit goal to form an impression (Winter & Uleman, 1984).

3 The manipulation conditions in the included studies were not always explicitly labeled as attitudinal or behavioral information in the original articles. Instead, we classified these conditions as attitudinal or behavioral information based on the definitions used in the present framework.

4 Although we comment on null effects within specific sections, our predictions compare across the effects of different conditions. Nonetheless, null effects are less informative than directional ones.

5 Li (2022) did not explicitly describe the manipulated information as attitudinal or behavioral information in the original article. Rather, we classified these conditions based on the definitions of attitudinal and behavioral information introduced in the present paper.

6 In Experiment 3 of Barnes and Shavitt’s (2023) paper, the relative influence of attitudinal and behavioral information differed between individuals with interdependent versus independent self-construal. Among participants with interdependent self-construal, attitudinal information exerted a significantly stronger influence than behavioral information. Among those with independent self-construal, there was no significant difference between the effects of attitudinal and behavioral information. To reflect these two patterns, we coded the experiment outcome in Table 2 as Attitude > = Behavior.
